# Using the Data Agreement Criterion to Rank Experts’ Beliefs

**DOI:** 10.3390/e20080592

**Published:** 2018-08-09

**Authors:** Duco Veen, Diederick Stoel, Naomi Schalken, Kees Mulder, Rens van de Schoot

**Affiliations:** 1 Department of Methods and Statistics, Utrecht University, 3584 CH 14 Utrecht, The Netherlands; 2 ProfitWise International, 1054 HV 237 Amsterdam, The Netherlands; 3 Optentia Research Focus Area, North-West University, Vanderbijlpark 1900, South Africa

**Keywords:** Bayes, Bayes factor, decision making, expert judgement, Kullback–Leibler divergence, prior-data (dis)agreement, ranking

## Abstract

Experts’ beliefs embody a present state of knowledge. It is desirable to take this knowledge into account when making decisions. However, ranking experts based on the merit of their beliefs is a difficult task. In this paper, we show how experts can be ranked based on their knowledge and their level of (un)certainty. By letting experts specify their knowledge in the form of a probability distribution, we can assess how accurately they can predict new data, and how appropriate their level of (un)certainty is. The expert’s specified probability distribution can be seen as a prior in a Bayesian statistical setting. We evaluate these priors by extending an existing prior-data (dis)agreement measure, the Data Agreement Criterion, and compare this approach to using Bayes factors to assess prior specification. We compare experts with each other and the data to evaluate their appropriateness. Using this method, new research questions can be asked and answered, for instance: Which expert predicts the new data best? Is there agreement between my experts and the data? Which experts’ representation is more valid or useful? Can we reach convergence between expert judgement and data? We provided an empirical example ranking (regional) directors of a large financial institution based on their predictions of turnover.

## 1. Introduction

In the process of scientific inference, the knowledge and beliefs of experts can provide vital information. Experts’ beliefs represent the current state of knowledge. It is desirable to be able to include this information in analyses or decision-making processes. This can be done by using the Bayesian statistical framework. In Bayesian statistics, there are two sources of information: prior knowledge and data [1,2,3]. The prior can be composed of expert knowledge [4,5,6]. However, deciding which expert yields the most appropriate information remains a critical challenge, for which we present a solution in this paper.

To be able to consider expert knowledge in Bayesian statistics, it must be represented in the form of a probability distribution. This can be done via a process called expert elicitation. Elicitation entails the extraction of expert knowledge and translating this knowledge into a probabilistic representation [5]. By using a probabilistic representation, we include both knowledge and (un)certainty of experts. However, experts are forced to use a representation system that belongs to the statistical realm. Therefore, it is essential that the elicitation process is carefully constructed so we do not introduce unnecessary and unjust bias.

The expression of expert knowledge in the form of a probability distribution is not merely based on statistical considerations. Forecasting without providing uncertainty estimates does not make sense, for, if we were certain, we would not predict but simply conclude future events to occur as they are inevitable. This would simply be a form of deductive logic and no discussion or disagreement based on the facts should be possible. Here, it is relevant to make the distinction between aleatory and epistemic uncertainty. Aleatory uncertainty is uncertainty due to randomness or chance, e.g., market volatility, whilst epistemic uncertainty is uncertainty due to a lack of knowledge. In practice, there is a blurred line between epistemic and aleatory uncertainty and the two can be seen as the ends on a spectrum, but, for the sake of argument, we shall make a clear distinction between the two here. In any case, if we can agree that, based on all the available information, there are still multiple outcomes possible, we have a situation in which we should start making forecasts including uncertainty estimates and probability distributions provide an excellent framework.

By collecting data and modeling the parameter of interest, we are able to gain an indication of the appropriate amount of uncertainty and the expected parameter value based on posterior distributions of interest in the model. In the limit, where we would not have epistemic uncertainty and all of the relevant background characteristics could be controlled for, any remaining residual variance in the model is the appropriate and correct amount of aleatory uncertainty. In practice, however, we do not have the perfect model and not all epistemic uncertainty can be ruled out, that is, we have not yet identified all relevant background characteristics. What we do have in practice are multiple experts with divergent beliefs on the relevant background characteristics. If we can evaluate their forecasts, including uncertainty, we can take more accurate forecasts as an indication of expertise on relevant aspects of the data generating process and we should let these experts guide us in identifying the relevant background characteristics. Moreover, if these knowledgeable experts can be identified and persuaded to share their insights with each other, they can start to learn from each other, the data and the appropriateness of assumptions underlying their forecasts. By expressing expert knowledge and data in the same framework, a learning process can start that has the potential to reduce uncertainty.

Once expert knowledge is elicited and data is collected, it is desirable to find a measure that naturally compares two pieces of information. The measure should assess the extent to which information from the data and expert knowledge resemble and conflict with each other. As the expert knowledge can be contained within a prior, it seems logical to assess the discrepancy or similarity of such a prior with respect to the data by means of a prior-data (dis)agreement measure. A desirable property for such a prior-data (dis)agreement measure would be to measure how one probability distribution diverges from a second probability distribution, rather than assessing the distance between two points estimates. The Data Agreement Criterion (DAC) [7] is based on Kullback–Leibler (KL) divergences [8] and therefore meets this desired property. KL divergence has previously been used in a related context to assess calibration and information scores of experts [9,10].

Prior-data (dis)agreement measures are currently used to evaluate, for example, the suitability of certain priors in the estimation of models or to uncover potential suitability problems with design, prior or both. Examples can be found in, for instance [11,12,13]. We found no previous use of prior-data (dis)agreement measures to rank experts. However, when we have two experts, some very interesting questions can already be answered, for instance: Which expert predicts the new data best? Is there agreement between my experts and the data? Which expert’s representation is more valid or useful? Can we reach convergence between expert judgement and data? Therefore, the main contribution of this paper will be to provide an application of prior-data (dis)agreement measures to expert ranking.

Other measures that answer similar questions on different theoretical basis can be found. For instance, Cohen’s kappa [14] could be used to assess inter-rater agreement, intraclass correlations [15] could be used to asses rater reliability [16] and Brier scores [17] can be used to asses discrepancy between experts’ estimated probability and actual outcomes [18]. These measures, however, do not account for the uncertainty of the experts over their provided estimates.

An alternative approach could be to use Bayes factors (BF) [19] based on marginal likelihoods. One could imagine different experts’ beliefs to be competing versions of models. When the differing views are expressed in different prior distributions, we could assess the likelihood of the data averaged across the prior distribution, which is what a marginal likelihood is [20]. This likelihood depends on the model structure, such as parametrization, or the set of probability distributions that is used as the model [21]. If we keep this set of probability distributions, the model, equal across the experts and the same data is used, the marginal likelihood provides an indication of which experts’ prior belief gives most probability to the data, and who is thus ranked most trustworthy. The BF, being a ratio of marginal likelihoods, could then provide us odds in favor of one expert’s beliefs over another’s. This approach warrants further comparison, which is given in Section 2.2.

In the remainder of this paper, we present the following work. We provide a detailed description of the DAC and explain why this measure is especially suitable to compare expert judgement and data. As the DAC currently determines the degree of prior-data (dis)agreement of one prior, we propose a straightforward adjustment of the statistic to allow the ranking of multiple sources of prior information, i.e., multiple experts’ beliefs. We discuss how Bayes factors could be used to rank experts based on their prior specifications. Finally, we provide an empirical example to show that the adapted DAC can be used to compare and rank several experts based on their beliefs and we compare this to using Bayes factors. In the empirical example, we rank experts from a large financial institution based on their predictions of new data concerning turnover. The empirical study in this article received approval from our internal Ethics Committee of the Faculty of Social and Behavioural Sciences of Utrecht University. The letter of approval can be found in the data archive for this study along with all other code and data, as far as contracts permit us, in order to ensure everything presented in this paper is reproducible. The data archive can be found on the Open Science Framework (OSF) webpage for this project at https://osf.io/u57qs.

## 2. Expert-Data (Dis)Agreement

Within this section, we discuss the DAC and the Bayes factor that are used to evaluate experts’ beliefs.

### 2.1. Data Agreement Criterion

Within this subsection, we provide a detailed and mathematical description of the DAC before proposing the adaptation that allows the ranking of multiple experts’ beliefs at the same time. The DAC is based on a ratio of KL divergences; therefore, we will first describe KL divergence [8].

#### 2.1.1. Kullback–Leibler Divergence

**Figure 1:**
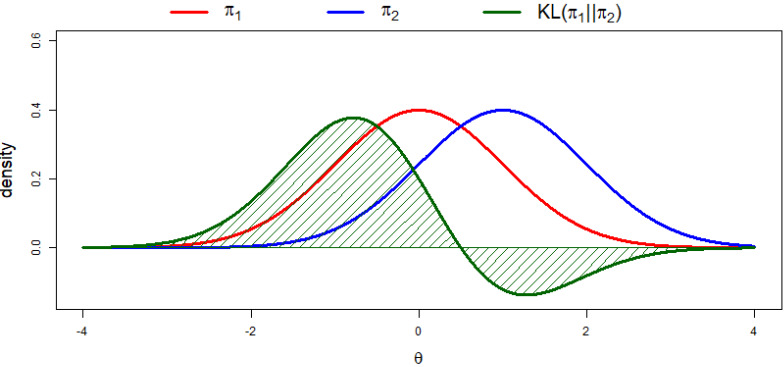
KL divergences between two normal distributions. In this example, $\pi_{1}$ is a standard normal distribution and $\pi_{2}$ is a normal distribution with a mean of 1 and a variance of 1. The value of the KL divergence is equal to the integral over the parameter space for the function. The green shaded area above the *x*-axis adds to the KL divergence and the green shaded area below the *x*-axis subtracts from the KL divergence.

The KL divergence describes measurements of informative regret, or, in other words, it measures the loss of information that occurs if the reference distribution ($\pi_{1}$) is approximated by another distribution ($\pi_{2}$). This loss of information or informative regret is expressed in a numerical value and the higher this value is, the more loss of information is present, i.e., the greater the discrepancy between the two distributions. The KL divergence is calculated by

(1) $$\mathrm{KL}(\pi_{1}||\pi_{2})=\int_{\Theta }\pi_{1}\left(\theta \right)\;\log\frac{\pi_{1}\left(\theta \right)}{\pi_{2}\left(\theta \right)}\;d\theta ,$$

where Θ is the set of all accessible values for the parameter θ, that is, its parameter space, $\pi_{1}\left(\theta \right)$ denotes the reference distribution and $\pi_{2}\left(\theta \right)$ denotes the distribution that approximates the reference distribution. In Figure 1, it can be seen what KL divergences between two normal distributions look like. The value of the KL divergence is equal to the integral over the parameter space for the function. The greater the discrepancy between the distributions, the larger the value of the integral. This also follows from Equation (1) because, if the two distributions are equal, then $\pi_{1}\left(\theta \right)/\pi_{2}\left(\theta \right)$ equals one everywhere. As log(1)=0, the integral, or loss of information, is equal to zero. To support understanding of the KL divergence, we build a shiny application that provides an interactive variant of Figure 1, which can be found via the OSF webpage at https://osf.io/u57qs.

If we are able to represent both the data and the expert knowledge in a distributional form, a discrepancy between the two can be expressed by the KL divergence between the two. As we might have multiple experts but only one source of data, it seems natural that the data be considered the reference distribution, which is approximated by the experts’ beliefs expressed as probability distributions. We will see in the following, where we elaborate on the details of this prior-data (dis)agreement measure developed by Bousquet [7], that this is indeed the case in the DAC.

#### 2.1.2. DAC

The DAC, as mentioned before, is a ratio of two KL divergences. A KL divergence provides an indication of the discrepancy between two distributions. KL divergence does not, however, have a natural cut-off value or threshold that can help us decide when a certain amount of loss of information would constitute prior-data disagreement. To be able to objectively conclude when prior-data disagreement exists, the DAC compares the loss of information that a certain prior has with respect to the data with the loss of information that a benchmark prior has with respect to the data. The KL divergence between the chosen prior and the data is the numerator in the ratio whilst the KL divergence between some benchmark prior and the data is the denominator in the ratio. A benchmark prior, denoted by $\pi^{J}\left(\theta \right)$, should be chosen such that the posterior distribution is completely dominated by the observed data y [22]. We denote such a posterior distribution by $\pi^{J}\left(\theta y\right)$ and use this as a representation of the data.

It is necessary to expand on the choice for the benchmark prior $\pi^{J}\left(\theta \right)$ and in relation to this the posterior distribution $\pi^{J}\left(\theta |y\right)$. Bousquet [7] follows the reasoning Bernardo provided in discussion with Irony and Singpurwalla [23] to see $\pi^{J}\left(\theta |y\right)$ as a non-subjective posterior that is representative of the situation that one’s prior knowledge was dominated by the data. In other words, $\pi^{J}\left(\theta |y\right)$ can be considered as a fictitious expert that is perfectly in agreement with the data, having no prior knowledge and being informed about the observations. $\pi^{J}\left(\theta |y\right)$ can be considered to be a reference posterior conveying the inferential content of the data [22].

If $\pi^{J}\left(\theta |y\right)$ is taken to be a reference posterior, this would implicitly support the choice of $\pi^{J}\left(\theta \right)$ such that it is a reference prior as originally developed by Bernardo [22], further developed by Berger and Bernardo, e.g., [24], described in Bernardo and Smith [25] and more formally worked out in Berger, Bernardo and Sun [26]. Reference priors are not the only possible choice for priors that convey in some sense minimal information or affect the information of the likelihood as weakly as possible [27]. An extensive overview can be found in Kass and Wasserman [28] and some notable options are Jeffrey’s priors [29,30] and maximum entropy priors [31] to which the reference priors reduce in specific cases [25].

One notable problem for using reference priors as a choice for $\pi^{J}\left(\theta \right)$ is that they often are improper priors [32] and KL divergences and thus the DAC are not well defined when one of the distributions is improper. An adaptation of the DAC could be used, however a choice for a more convenient prior that is proper and leads to a posterior $\pi^{J}\left(\theta |y\right)$ closely resembling a reference posterior seems reasonable [7].

Now taking $\pi^{J}\left(\theta |y\right)$ as the reference posterior, $\pi^{J}\left(\theta \right)$ as the benchmark prior and the data y, the DAC for a chosen (expert) prior, denoted by π(θ), can be expressed by

(2) $$\mathrm{DAC}=\frac{\mathrm{KL}[\pi^{J}(.|y)||\pi ]}{\mathrm{KL}[\pi^{J}(.|y)||\pi^{J}]},$$

following the notation of Bousquet.

The benchmark, being an uninformative prior, should by definition not be conflicting with the data and therefore serves as a good reference point to determine if a certain amount of loss of information can be considered to be relevant. If a prior conflicts less with the data than the benchmark does, we should consider the prior to be in prior-data agreement. If a prior conflicts more with the data than the benchmark prior does, we do consider the prior to be in prior-data disagreement. Hence, if the DAC > 1, we conclude prior-data disagreement because the KL divergence of the prior is larger than the KL divergence of the benchmark prior; otherwise, we conclude prior-data agreement.

**Figure 2:**
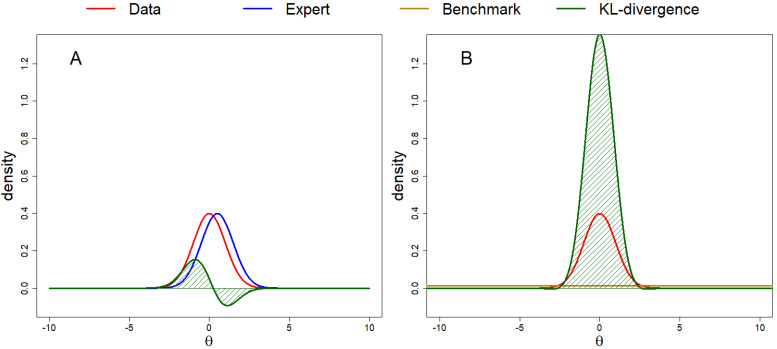
Calculating the DAC. In this example, $\pi^{J}\left(\theta |y\right)$ is a standard normal distribution, π(θ) is a normal distribution with a mean of 0.5 and a variance of 1 and $\pi^{J}\left(\theta \right)$ is a normal distribution with a mean of 0 and a variance of 900. The DAC < 1, thus prior-data agreement is concluded.

To illustrate the calculation of the DAC, we provide a numerical example together with a visual representation that can be found in Figure 2. Consider the case in which π^J^ (θ│**y**) is the is the N(0, 1) density, π(θ) is the N(0.5, 1) density and $\pi^{J}\left(\theta \right)$ is the N(0, 900) density. The DAC is then calculated by taking the ratio of the following two KL divergences, Figure 2A; $\mathrm{KL}[\pi^{J}(.|y)||\pi ]=0.125$ and Figure 2B; $\mathrm{KL}[\pi^{J}(.|y)||\pi^{J}]=2.902$, such that DAC = 0.125/2.902 = 0.043. The DAC < 1, thus we conclude prior-data agreement, and π(θ) is a better approximation of $\pi^{J}(\theta |y)$ than .

#### 2.1.3. Extension to Multiple Experts

**Figure 3:**
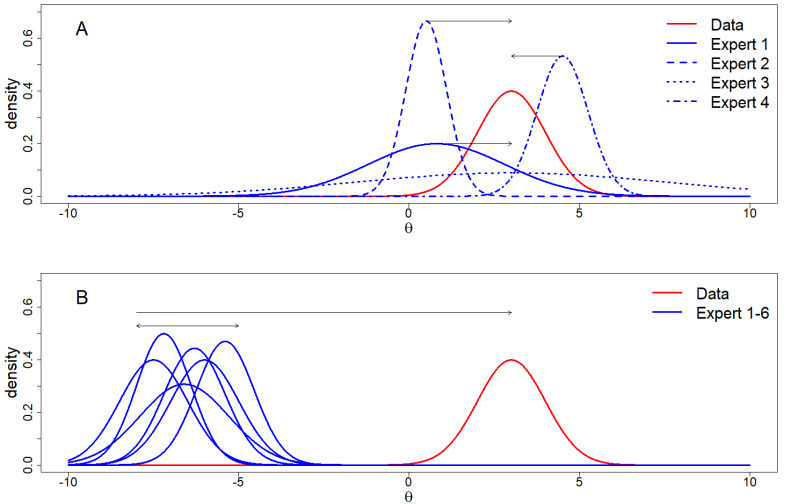
Scenarios in which there are multiple experts and one source of data. (**A**) shows experts differing in prediction and (un)certainty, all (dis)agreeing to a certain extent with the data; (**B**) shows a scenario in which all experts disagree with the data, which results in the question of which of the sources of information is correct.

The DAC, as described in the section above, determines the degree of prior-data (dis)agreement for a single prior that is to be evaluated. However, when we have multiple experts that each hold their own beliefs and we express each of these in the form of a probability distribution, we can ask some interesting questions. In Figure 3, we see some examples of situations that we could encounter. In Figure 3A, we see a situation in which experts differ in their predictions and their (un)certainty. The question that arises from the situation in Figure 3A is which of these predictions best approximates the information that the data provides us? Figure 3B shows a scenario in which the experts are predicting similar to each other but all differ with respect to the data. The question that arises from the situation in Figure 3B is which of the two is correct, the data or the experts?

To be able to answer these types of questions, we need to extend the DAC to incorporate multiple experts’ priors, which are to be evaluated against the same posterior distribution, reflecting the data, and the same benchmark prior. The DAC thus needs to become a vector of length D resulting in

(3) $${\mathrm{DAC}}_{d}=\frac{\mathrm{KL}[\pi^{J}(.|y)||\pi_{d}]}{\mathrm{KL}[\pi^{J}(.|y)||\pi^{J}]},$$

where the subscript d denotes the different input for D experts so ${\mathrm{DAC}}_{d}={\mathrm{DAC}}_{1},\ldots ,{\mathrm{DAC}}_{D}$ and $\pi_{d}\left(\theta \right)=\pi_{1}\left(\theta \right),\ldots ,\pi_{D}\left(\theta \right)$. This extension of the KL divergence in which not one but a vector of models are entered to be compared with the preferred model is straightforward and has previously been described in the context of the Akaike Information Criterion (AIC) [33,34].

#### 2.1.4. Influence of the Benchmark

The choice for a specific benchmark can influence the results of the ${\mathrm{DAC}}_{d}$. Bousquet [7] suggests that, in applied studies, the availability of a convenient or intuitive prior for the benchmark seems reasonable. However, it is important to realize that the choice for a benchmark prior does influence the results of the analysis in the sense that the cut-off value for determining prior-data disagreement will shift as the KL divergence between $\pi^{J}(\theta |y)$ and $\pi^{J}\left(\theta \right)$ changes. However, as long as the benchmark prior is an uninformative prior in the sense that the posterior distribution is dominated by the data, $\pi^{J}(\theta |y)$ will remain largely unchanged. This ensures that the ${\mathrm{DAC}}_{d}$ has the good property that when multiple experts are compared their ranking does not change dependent on which uninformative benchmark is chosen. This follows from the stability of $\pi^{J}\left(\theta |y\right)$, which ensures that the KL divergences between $\pi^{J}(\theta |y)$ and $\pi_{d}\left(\theta \right)$ are stable. Different choices for $\pi^{J}\left(\theta \right)$ do change the KL divergence in the denominator and therefore shift the prior-data disagreement boundary.

**Figure 4:**
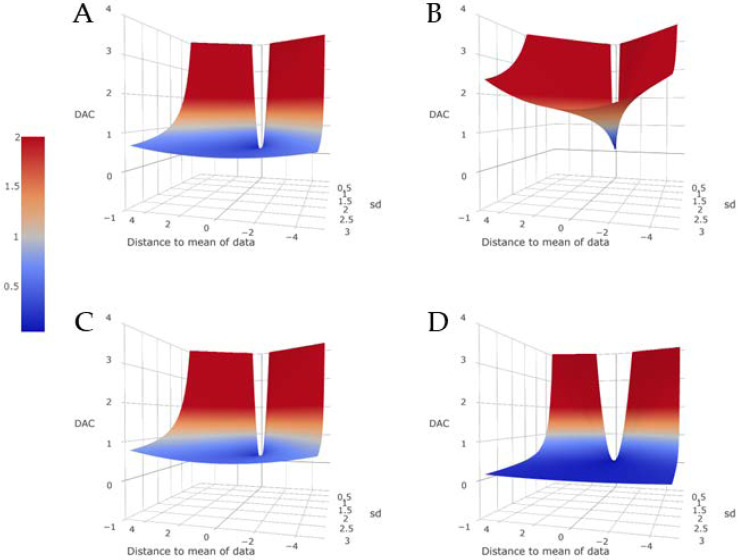
The effect on the behavior of the ${\mathrm{DAC}}_{d}$ for different choices for benchmark priors. All panels use the same data (*N* = 100) from a standard normal distribution and the same variations for $\pi_{d}\left(\theta \right)$ which are the normal distribution for which the parameters for the mean and standard deviation are given on the *x*-axis and *y*-axis of the panels. In (**A**), the benchmark is the N(0, 10,000) density; in (**B**), the N(0, 1) density; in (**C**), the U(−50, 50) density and in (**D**), the N(5, 0.5) density.

Concerning the benchmark, it is useful to note that the benchmark need not be restricted to an uninformative prior, but using an informative prior changes the interpretation and behavior of the DAC. When $\pi^{J}\left(\theta \right)$ is informative, $\pi^{J}(\theta |y)$ is sensitive to the specification of $\pi^{J}\left(\theta \right)$ and the KL divergence between $\pi^{J}(\theta |y)$ and $\pi_{d}\left(\theta \right)$ need no longer be stable, potentially influencing the ranking of the experts. To show the above described behavior visually, we present the results of a simulation study in Figure 4. We show four different conditions, that is, four different choices for benchmark priors, to illustrate the change in behavior for the DAC_d_. In all four situations, we use the same data, **y**, which is a sample of 100 from a standard normal distribution with a sample mean $\bar{y}$. $\pi_{d}\left(\theta \right)$ is the $N\left(\mu_{0},\;\sigma_{0}^{2}\right)$ density and we show the ${\mathrm{DAC}}_{d}$ values for $\mu_{0}=\bar{y}-4,\;\ldots ,\;\bar{y}+4$ and $\sigma_{0}=0.1,\ldots ,\;3$. The four panels show different conditions for the benchmarks such that, in Figure 4A, it is the N(0, 10,000) density, in Figure 4B, the N(0, 1) density, in Figure 4C, the U(−50, 50) density and in Figure 4D the N(5, 0.5) density. It can be seen that, for the two uninformative priors in Figure 4A,C, the behavior of the ${\mathrm{DAC}}_{d}$ is stable. We would expect to draw the same conclusions and rank experts in the same way independent of the choice of either benchmark. However, when we specify an informative benchmark such as in Figure 4B,D, we see that both the behavior of the ${\mathrm{DAC}}_{d}$ and the determination of prior-data (dis)agreement shift. In Figure 4B, an informative and accurate benchmark leads almost invariably to concluding prior-data disagreement for $\pi_{d}\left(\theta \right)$. In Figure 4D, the informative but inaccurate benchmark leads us to conclude prior-data disagreement only if $\pi_{d}\left(\theta \right)$ is in the wrong location and has a very small variance.

The simulation study presented in Figure 4 shows that the choice for a certain benchmark can influence your results, so, even if a convenient or intuitive prior seems reasonable, it should be carefully chosen. Researchers should be aware that their ranking is stable as long as an uninformative prior is chosen, but it might not be if the benchmark prior contains information.

### 2.2. Comparison to Ranking by the Bayes Factor

In order to develop a good understanding of the behavior of the DAC for expert ranking, this section will provide a comparison to expert ranking using Bayes factors, that is, by ranking experts on the marginal likelihood resulting from their prior. First, we provide a mathematical description of the Bayes Factor (BF), which is a ratio of marginal likelihoods. Then, the influence of the benchmark prior will be discussed, followed by a comparison of expert ranking via Bayes Factors to expert ranking through the DAC.

#### 2.2.1. Marginal Likelihood

For a model *M* and observed data **y**, denote the likelihood f(y|θ) and prior π(θ) such that the posterior distribution

(4) $$\pi \left(\theta |y\right)=\frac{f\left(y|\theta \right)\pi \left(\theta \right)}{\int_{\Theta }f\left(y|\theta \right)\pi \left(\theta \right)d\theta }.$$

The denominator on the right-hand side of Equation (4) is the marginal likelihood m(y), sometimes called the evidence. The marginal likelihood can be thought of as the probability of the data averaged over the prior distribution [20]. As the probability of the data is dependent on the model, which is the set of probability distributions that is used [21], the marginal likelihood is influenced by the choice of model *M,* the data **y** and the prior π(θ). If we have *d* experts and we keep *M* and **y** equal across experts, the only difference in $m_{d}\left(y\right)$ arises from the different specified priors $\pi_{d}\left(\theta \right)$. We could thus differentiate between experts by assessing the probability of the data averaged across their specified prior beliefs.

#### 2.2.2. Bayes Factor

The BF can be used to compare the marginal likelihoods for the different experts, $m_{d}\left(y\right)$, such that, for example,

(5) $${\mathrm{BF}}_{1d}=\frac{m_{1}\left(y\right)}{m_{d}\left(y\right)}$$

provides the odds in favor of some model $M_{1}$, versus model $M_{d}$, the model that has the prior provided by expert d. As the set of probability distributions that is used and the data y are the same between experts, this essentially provides the odds in favor of the prior $\pi_{1}\left(\theta \right)$ versus prior $\pi_{d}\left(\theta \right)$. Similarly, experts could be compared directly. It is well known that the BF is sensitive to the specification of different priors via the marginal likelihoods that are used [19,20,21,35]. Liu and Aitkin [20] note that this is not necessarily undesirable. Moreover, in our case, this property is essential in allowing the evaluation of the relative merit of the experts’ beliefs that are specified in the form of prior probability distributions.

#### 2.2.3. Benchmark Model

The BF allows us to compare the odds in favor of one expert over another but neither the individual marginal likelihoods based on expert priors nor the ratios provide us with an assessment of the inherent appropriateness of the prior in terms of (dis)agreement between the prior and the data. As with the DAC, we could imagine taking a benchmark prior $\pi^{J}\left(\theta \right)$ that serves as a reference point such that the marginal likelihood is $m^{J}\left(y\right)$. If we take

(6) $${\mathrm{BF}}_{\mathrm{Jd}}=\frac{m^{J}\left(y\right)}{m_{d}\left(y\right)}$$

and if ${\mathrm{BF}}_{\mathrm{Jd}}<1,$ we would favor the model using the expert prior and conclude agreement with the data and, if ${\mathrm{BF}}_{\mathrm{Jd}}>1,$ we would favor the model using the benchmark prior and conclude disagreement with the data.

However, we run into the same issue as with the KL divergences because the marginal likelihood is ill-defined if improper priors are used [19,20,21]. Thus, again, reference priors [22] are not suitable for use in this context. Raftery suggests using a reference set of proper priors [36] and both Kass and Raftery [19] and Liu and Aitkin [20] suggest conducting a sensitivity analysis in any case. To keep the comparison between the ${\mathrm{BF}}_{\mathrm{Jd}}$ and the ${\mathrm{DAC}}_{d}$ straightforward, we will use the same benchmark prior $\pi^{J}\left(\theta \right)$ in both situations. As both ${\mathrm{BF}}_{\mathrm{Jd}}$ and ${\mathrm{DAC}}_{d}$ are sensitive to the choice for $\pi^{J}\left(\theta \right)$, a sensitivity analysis will be included in the empirical part of this paper. Note that this sensitivity is most evident when using these tools as a prior-data conflict criterion, as the expert rankings will generally remain unchanged for different uninformative benchmark priors.

### 2.3. DAC Versus BF

Burnham and Anderson state that the BF is analogous to the information-theoretic evidence ratio [34], for instance, the DAC. If we directly compare two experts with a BF, we would obtain odds favoring one expert over another and if we compare the KL divergences between two experts, we could state that one expert has a certain amount of times the loss of information in relation to another. Despite the analogy, they are also inherently different. This is most clearly seen when we compare the alternative form of the DAC from Bousquet [7], which is given in our case by

$${\mathrm{DAC}}_{2,d}^{J}=\frac{m^{J}\left(y\right)}{m_{d}\left(y\right)}\exp\left\{\mathrm{KL}[\pi^{J}\left(\cdot |y\right)||\pi_{d}\left(\cdot |y\right)]\right\}={\mathrm{BF}}_{\mathrm{Jd}}\exp\left\{\mathrm{KL}[\pi^{J}\left(\cdot |y\right)||\pi_{d}\left(\cdot |y\right)]\right\}.$$

Therefore, the difference between the DAC and BF can clearly be seen to be the fact that the DAC has an additional term which multiplies the BF by $\exp\left\{\mathrm{KL}[\pi^{J}\left(\cdot |y\right)||\pi_{d}\left(\cdot |y\right)]\right\}$, the KL divergence between the reference posterior and the posterior from expert *d*. This additional term is desirable, as it penalizes experts who are overly certain more harshly than the BF would.

To illustrate this, consider the following limiting case. Imagine an expert who believes that they are infinitely certain about the future. This expert should then specify their prior in the form of a Dirac delta function $\delta_{\theta_{0}}\left(\theta \right)$, also called the degenerate distribution on the real line, which has density zero everywhere for θ except for $\theta_{0}$ where it has infinite density [37]. Moreover, the delta function actually integrates to one and in that sense is a proper prior which can also be viewed as an infinitely narrow Gaussian $\delta \left(\theta -\theta_{0}\right)=\underset{\sigma \to 0}{\lim}N(\theta |\theta_{0},\;\sigma^{2})$ [38]. Now, if an expert states their prior belief in the form of a delta function and $\theta_{0}$ coincides with a region of θ where the likelihood f(y|θ)>0, both the marginal likelihood and $\mathrm{KL}[\pi^{J}\left(\cdot |y\right)||\delta_{\theta_{0}}\left(\cdot \right)]$ will become infinite. The meaning could, however, not differ any more. The marginal likelihood suggests that this expert is the best possible expert, whilst the KL divergence suggests that there is no worse expert. Although this scenario is quite extreme, van de Schoot, Griffioen and Winter [39] did encounter such an expert in their elicitation endeavors.

## 3. Empirical Example

To show that the ${\mathrm{DAC}}_{d}\;$ can be used to evaluate and rank several experts based on their beliefs, we conducted an empirical study. The team that participated consisted of 11 experts, 10 regional directors and one director. All were eligible to be included in the study. Seven experts were randomly invited to participate in the research; if any of the selected experts did not want to participate, they were classified as not selected in the research. In this way, we avoided the possibility of group pressure to participate. In the end, four out of the seven selected experts participated in an elicitation. The experts (*D* = 4) provided forecasts concerning average turnover per professional in the first quarter of the year 2016. The (regional) directors are considered experts in knowledge concerning market opportunities, market dynamics and estimating the capabilities of the professionals to seize opportunities. Based on these skills, we expected that they could predict the average turnover per professional in the entire country in the first quarter of 2016. All information related to the empirical study can be found on the OSF webpage for this paper at https://osf.io/u57qs.

### 3.1. Elicitation Procedure

To get the experts to express their beliefs in the form of a probability distribution, we make use of the Five-Step Method [40]. To encapsulate the beliefs of the expert, the Five-Step Method actively separates two elements of the knowledge of the expert: tacit knowledge of the expert and their (un)certainty. In step one, a location parameter is elicited from the expert. This location parameter captures the tacit knowledge of the expert. To verify that the representation of the beliefs is accurate, step two is the incorporation of feedback implemented through the use of elicitation software. Experts can accept the representation of their beliefs or adjust their input. In step three, the (un)certainty of the experts is obtained and represented in the form of a scale and shape parameter. Step four is to provide feedback using elicitation software to verify the accurate representation of the expert’s (un)certainty, which they can either accept or they can adjust their input until the representation is in accordance with their beliefs. The fifth step is to use the elicited expert’s beliefs, in this case to determine their DAC score.

The experts first performed a practice elicitation for their own sales team before moving on to the whole country. The practice run enabled them to acquaint themselves with the elicitation procedure and software we used. The elicited distributions were restricted to be skewed normal distributions such that $\pi_{d}\left(\theta \right)$ are $SN\left(\mu_{0},\sigma_{0}^{2},\gamma_{0}\right)$ densities where subscript *d* denotes expert *d* = 1, …, *D*, $\mu_{0}$ denotes the prior mean, $\sigma_{0}^{2}$ denotes the prior variance and $\gamma_{0}$ denotes the prior skewness. The shape parameter $\gamma_{0}$ is based on a general method for the transformation of symmetric distributions into skewed distributions as described by Equation (1) in Fernandez and Steel [41]. Table 1 provides an overview of the elicited distributions for the four experts in this empirical study. The distributions are based upon transformed data to avoid revealing business-sensitive information.

### 3.2. Ranking the Experts

The predictions of the experts concerned the average turnover per professional (*N* = 104). The benchmark is the U(0, 5) density. A uniform distribution was chosen for the normal model in line with the prior used by Bousquet [7] in his Example 1 concerning a normal model. The lower bound of 0 arises out of the natural constraint that negative turnover will not occur, the upper bound of 5 was considered as a value that could not be attained, yet this number is to some extent arbitrary and a sensitivity analysis was conducted to investigate the impact of the choice for $\pi^{J}\left(\theta \right)$. With regard to the desired minimal influence of $\pi^{J}\left(\theta \right)$ on $\pi^{J}\left(\theta |y\right)$, in our case, the reference posterior can be analytically calculated (see Yang and Berger [32]). The KL divergence for approximating the reference posterior with $\pi^{J}\left(\theta |y\right)$ was 0.00016, which we considered to be negligible.

**Figure 5:**
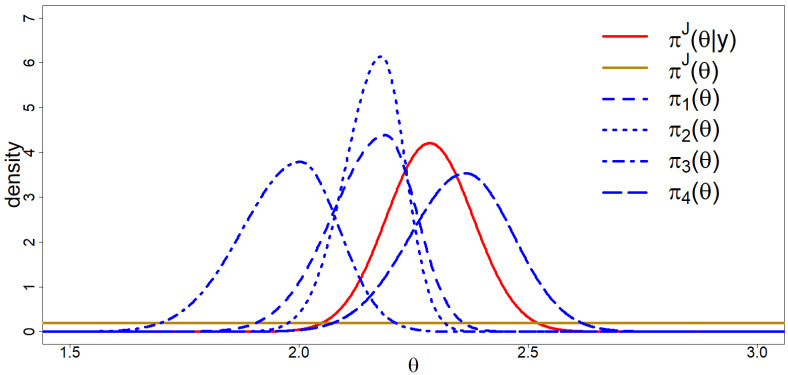
Visual presentation of all relevant distributions for the empirical study; $\pi_{d}\left(\theta \right)$, $\pi^{J}\left(\theta \right)$ and $\pi^{J}(\theta |y)$.

**Figure 6:**
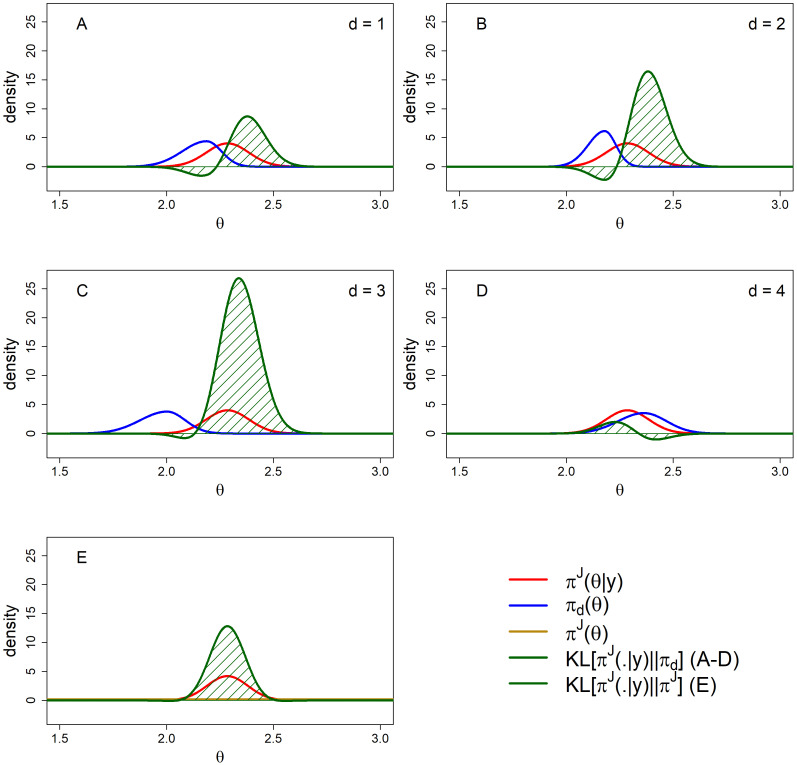
All KL divergences for $\pi_{d}\left(\theta \right)$ (**A**–**D**) and $\pi^{J}\left(\theta \right)$ (**E**) with $\pi^{J}(\theta |y)$ as the distribution that is to be approximated. (**A**) is for expert one; (**B**) for expert two; (**C**) for expert three and (**D**) for expert four.

We obtained the posterior distribution $\pi^{J}\left(\theta |y\right)$ using the rjags R-package [42], such that $\pi^{J}\left(\theta |y\right)$ is the $N\left(\mu_{1},\sigma_{1}^{2}\right)$ density where $\mu_{1}$ denotes the posterior mean and $\sigma_{1}^{2}$ denotes the posterior variance. We used four chains of 25,000 samples after a burn-in period of 1000 samples per chain. Visual inspection and Gelman–Rubin diagnostics [43] did not point towards problems with convergence of the chains and inspection of the autocorrelation plots showed no issues concerning autocorrelation. To compute the marginal likelihoods and BF, we used the R-Package rstan [44] with four chains of 1000 samples after burn-in to obtain the posterior distributions and we used the bridgesampling R-package [45] to obtain the marginal likelihoods and BF. For more details, see the data archive on the OSF webpage. Table 2 displays KL divergences, ${\mathrm{DAC}}_{d}$ scores and ranking, marginal likelihoods and ${\mathrm{BF}}_{\mathrm{Jd}}$ scores and ranking. Figure 5 visually presents all relevant distributions concerning the empirical study. Figure 6 panels A through E visually present all KL divergences from Table 2. Table 3 presents the results for the sensitivity analysis for different choices for π^J^ (θ) an and Table 4 allows for a comparison between experts without reference to any benchmark $\pi^{J}\left(\theta \right)$.

The results of Table 2 show that expert four provided the best prediction out of the experts, when using both the ${\mathrm{DAC}}_{d}{\;\mathrm{and}\;\mathrm{the}\;\mathrm{BF}}_{\mathrm{Jd}}.$ Experts one and two provided similar predictions concerning their tacit knowledge; they expected almost the same value for the location parameter; however, expert one was less certain about this prediction (see Table 1). As the prediction of the location was not entirely correct, the increased uncertainty of expert one means that this expert provided more plausibility to the regions of the parameter space that were also supported by the data. Here we see the difference between ${\mathrm{DAC}}_{d}$ and the ${\mathrm{BF}}_{\mathrm{Jd}}$ arise as discussed in Section 2.3. Overconfidence is penalized more severely by the ${\mathrm{DAC}}_{d}$ and as such the conclusion on which expert would be preferred changes between experts one and two depending on which measure you use. When we look at the ${\mathrm{DAC}}_{d}$, in the case when $\pi^{J}\left(\theta \right)$ is the U(0, 5) density, the additional penalization of the overconfidence even causes a different conclusion between experts one and two, namely, expert one is in prior-data agreement and expert two is in prior-data disagreement. For the ${\mathrm{BF}}_{\mathrm{Jd}}$ both are concluded to be in agreement with the data. Expert three provided a prediction that, to a large extent, did not support the same parameter space as the data. In fact, expert three provides a lot of support for regions of the parameter space that the data did not support. The discrepancy between expert three and the data was of such proportions that, besides expert two, we also concluded a prior-data disagreement to exist for expert three. If we had no information beforehand, except knowing the region within which the average turnover per professional could fall, we would have lost less information than by considering the predictions of experts two and three. The ${\mathrm{BF}}_{\mathrm{Jd}}$ differs from the ${\mathrm{DAC}}_{d}$ in the sense that when $\pi^{J}\left(\theta \right)$ is the U(0, 5) density, the benchmark only outperforms expert 3.

From the sensitivity analyses of Table 3 we can find that the reference posterior remains quite stable and therefore the KL divergences for the experts do not change substantially; however, the changing KL divergence for the benchmark would shift the prior-data disagreement boundary. When $\pi^{J}\left(\theta \right)$ was the $N\left(0,\;10^{3}\right)$ or $N\left(0,\;10^{4}\right)$ density, expert three would no longer be in prior-data conflict, whilst prior-data disagreement for expert two was only concluded if $\pi^{J}\left(\theta \right)$ was the U(0, 5) density. For the BF changing the benchmark also shifts the prior-data (dis)agreement boundary arbitrarily. In this case our decisions on prior-data (dis)agreement would only change for the $N\left(0,\;10^{4}\right)$ prior, where expert 4 would no longer be in prior-data disagreement. The sensitivity analysis showed that decisions on prior-data (dis)agreement might not be entirely reliable, whilst the ranking of experts remained stable.

Table 4 shows the results when we only compare experts on their KL divergences and their marginal likelihoods and we omit the benchmarks. We see the difference between the BF and the KL divergence ratios when we compare experts one and two. The differences arise from the more severe penalization of overconfidence by KL divergences compared to BF, as discussed in Section 2.3. Using KL divergence ratios we concluded that expert two had twice the amount of loss of information, whilst the BF even favors expert two over expert one with odds of 1.22.

The results of the empirical study show a slight difference in the conclusions with regard to the ranking of the experts depending on which measure we used, ${\mathrm{DAC}}_{d}$ or ${\mathrm{BF}}_{\mathrm{Jd}}$. Both measures select the same expert as being the best. If decisions should be made concerning average turnover per professional, decision makers would be wise to consult expert four, as this expert seemed to have the best knowledge of the underlying factors driving these results.

## 4. Discussion

In this paper, we use both the BF and the DAC to rank experts’ beliefs when they are specified in the probabilistic form of prior distributions. When comparing the BF and the DAC, the limiting case example of Section 2.3 springs to mind. In the introduction, we stated that forecasting without specifying uncertainty would not make sense to us and, in that light, we would prefer to use a measure that would classify doing so as undesirable behavior and punish this extreme case. An example of this behavior can be seen in the empirical example where while using the BF we would favor expert two over expert one, however whilst using KL divergences, we would favor expert one over expert two.

The sensitivity analysis in the empirical example, however, also highlighted some undesirable characteristics of the DAC for our context, namely the sensitivity to different choices for $\pi^{J}\left(\theta \right)$. In the context of ranking experts, it can make sense to drop the association between $\pi^{J}\left(\theta \right)$ and $\pi^{J}(\theta |y)$. $\pi^{J}(\theta |y)$ can remain a reference posterior and as such represent the characteristics of **y**. $\pi^{J}\left(\theta \right)$ can either be omitted or be specified such that it is meaningful. If $\pi^{J}\left(\theta \right)$ is omitted, we do not have a reference point for (dis)agreement; however, if arbitrarily chosen benchmarks shift this reference point, it hardly has any meaning. Without a benchmark, experts can still be compared with each other in terms of ratios of loss of information, as presented in Table 4. However, if π^J^ (θ) is meaningful, one could imagine, for instance, a gold standard that is used in a forecasting situation; we can assess experts’ beliefs in relation to this meaningful benchmark and see if they outperform this benchmark. If the association between $\pi^{J}\left(\theta \right)$ and $\pi^{J}\left(\theta |y\right)$ is dropped, we can specify informative benchmarks without the adverse effects of changing $\pi^{J}\left(\theta |y\right)$ and thereby the divergences between $\pi^{J}\left(\theta |y\right)$ and $\pi_{d}\left(\theta \right)$. Moreover, specifying informative benchmarks requires elaboration of the rationale behind the choice, thus enhancing trust in the conclusions if a sensitivity analysis shows different priors representing similar information that leads us to the same conclusions.

One of the reasons for the sensitivity of the DAC to different choices for $\pi^{J}\left(\theta \right)$ can be seen by comparing the KL divergences of expert one and two of the empirical example. As a referee pointed out to us, KL divergences are tail sensitive and this can be seen in this comparison. Expert one is a little more uncertain and as such the tail of $\pi_{1}\left(\theta \right)$ overlaps somewhat more with $\pi^{J}\left(\theta |y\right)$ than the tails of $\pi_{2}\left(\theta \right)$. This leads to half the loss of information. One could deem this tail sensitivity to be undesirable and, with differently shaped prior distributions, this problem might become more pronounced. If it is deemed undesirable, one could favor using the BF, which actually favors expert two with odds of 1.22 over expert 1. Alternatively, an interesting area for future research could be to investigate the use of alternative divergence measures. A good starting point for finding alternative measures can be found in the Encyclopedia of Distances by Deza and Deza [46].

In the current paper, we followed Bousquet [7] and used KL divergences and this raises two important methodological issues; see Burnham and Anderson [34] for an elaborated discussion. First, the reference model should be known. Second, the parameters should be known for the model that is evaluated, i.e., the formalized expert prior. The issues make the KL divergence a measure that, according to some, for instance Burnham and Anderson [34], cannot be used for real world problems and previously led to the development of the AIC [33], which uses the relative expected KL divergence. The AIC deals with the two issues by taking the reference model as a constant in comparing multiple models and using the maximum likelihood estimates for the parameters of the models to be evaluated, introducing a penalty term for the bias that this induces.

We conclude that we can use the KL divergence in the context of the ${\mathrm{DAC}}_{d}$ and with the following reasoning. We define $\pi^{J}(\theta |y)$ to be the reference distribution as it reflects a fictional expert that is completely informed by the data and thus it is known. In the case of the empirical example, the data is even the true state of affairs, i.e., the actual realizations of the turnover for each professional. Concerning the parameter for the models to be evaluated, $\pi_{d}\left(\theta \right)$ should reflect the exact beliefs of the experts. We use the Five-Step Method [40] which incorporates feedback at each stage of the elicitation, ensuring that experts confirm that their beliefs are accurately represented by the location, shape and scale parameters. We acknowledge that whether the parameters represent exactly an expert’s beliefs cannot be known, but we feel confident that the procedure we use at least aims to obtain very accurate representations. As experts can continue to adapt their input until they are satisfied with the representation of their beliefs, this should overcome problems with the second issue.

While we use $\pi^{J}(\theta |y)$, and thus know the reference distribution, and we firmly believe that we properly represent the experts’ beliefs, it seems highly implausible that a DAC score of 0 can be attained. It is unlikely that, in predicting future events, one estimates precisely the optimal location and exactly the optimal amount of uncertainty.

Although a priori specification of optimal uncertainty is unlikely, we are able to gain an indication of the appropriate amount of uncertainty a posteriori. $\pi^{J}(\theta |y)$ provides an excellent indication of the appropriate uncertainty. Given that one had no knowledge beforehand and is rationally guided by the data, following probabilistic reasoning, one arrives at the posterior belief represented by $\pi^{J}(\theta |y)$ [7,23]. The posterior described the range of values that would have been plausible given this information. This indication is, however, conditional on the fact that the data provide an accurate representation of the state of affairs.

Given that we can attain information on the expected value for the parameter of interest, the appropriate amount of uncertainty and the quality of the approximation by each expert, we can start a learning process. By sharing the reasons behind the choices they made, experts can learn from one another as evidence shows which reasoning leads to the most accurate predictions. The data can inform the experts so that they can adjust their estimates and uncertainty. Through this evaluation, expertise can increase and in the long run convergence should be reached between both different experts’ predictions and between the experts and the data. When this convergence is reached, this indicates that at least part of the epistemic uncertainty is eliminated and we have a better understanding of the data generating processes and are better able to make an informed decision. Note that, if we wish to incorporate the relevant factors that are identified by the experts, these should be included in the model so that part of the posterior uncertainty about our parameter can be explained. The explained variance can be seen as a reduction of the epistemic uncertainty or learning effect.

In the empirical example, we can already see some opportunities for learning. For example, expert three misestimated the location of the parameter, which indicates, at least to some extent, faulty or missing tacit knowledge. By starting a dialogue with the other experts, he or she could learn why they all estimated the average turnover per professional to be higher. Expert one and two had almost identical predictions concerning the location, but expert one expressed more uncertainty. Perhaps this indicated more acknowledgement of epistemic uncertainty; a dialogue could shed more light on the differences in choices of expert one and two. Our empirical example contains just four experts, but the methods used are easily scalable to include more experts, with only additional elicitation efforts required. Including more experts can result in more opportunities for learning.

Concerning the appropriateness of the ranking that is obtained using the ${\mathrm{DAC}}_{d}$, we have the following to add. One could argue that perhaps the sample entails extreme data. However, even if this is true, the experts should have considered the data to be plausible, for it did occur. Thus, if an expert exhibits large KL divergence with $\pi^{J}(\theta |y)$, this expert simply did not expect that these data were likely or plausible. By incorporating (un)certainty in the evaluation, the ${\mathrm{DAC}}_{d}$, or KL divergences if a benchmark is omitted, produces the required behavior to fairly compare experts’ beliefs. Given that it is appropriate to take uncertainty into account, a prior can be over-specific such that it does not adhere to the principles underlying the data generating mechanism. KL divergences reward the specification of an appropriate amount of uncertainty and penalize overconfidence.

To conclude this discussion, we state recommendations for researchers facing similar problems:

- Use ${\mathrm{DAC}}_{d}$ instead of BF.
- Specify $\pi^{J}(\theta |y)$ such that it serves as a reference posterior and drop the association between $\pi^{J}(\theta |y)$ and $\pi^{J}\left(\theta \right)$.
- Consider whether a meaningful benchmark can be determined. If not, only use $\mathrm{KL}[\pi^{J}(.|y)||\pi_{d}]$ and compare experts with each other and not with a benchmark.
- Carrying out a sensitivity analysis is always recommendable, even more so if benchmarks are used.

## Figures and Tables

**Table 1:** The values of the hyper parameters of $\pi_{d}\left(\theta \right)$ for the empirical study.

	$\mu_{0}$	$\sigma_{0}$	$\gamma_{0}$
Expert 1	2.15	0.09	0.78
Expert 2	2.16	0.07	0.82
Expert 3	1.97	0.11	0.82
Expert 4	2.35	0.11	0.94

**Table 2:** KL divergences, ${\mathrm{DAC}}_{d}$ scores and ranking, marginal likelihoods and ${\mathrm{BF}}_{\mathrm{Jd}}$ scores and ranking, for the experts’ priors and the benchmark prior. Note that marginal likelihoods are reported and not the log marginal likelihoods.

	KL Divergence	DAC_d_	DAC_d_ Ranking	$m_{d}\left(y\right)\;\&\;m^{J}\left(y\right)$	$BF_{Jd}$	$BF_{Jd}\;\mathrm{Ranking}$
Expert 1	1.43	0.56	2	5.57 × 10^−68^	0.21	3
Expert 2	2.86	1.12	3	6.82 × 10^−68^	0.17	2
Expert 3	5.76	2.26	4	2.19 × 10^−69^	5.31	4
Expert 4	0.19	0.07	1	1.72 × 10^−67^	0.07	1
Benchmark	2.55	-	-	1.16 × 10^−68^	-	-

**Table 3:** Sensitivity analysis for different choices for $\pi^{J}(\theta )$. Densities are given in the columns. The KL divergences and marginal likelihood $m^{J}\left(y\right)$ are presented in the rows. $m_{d}\left(y\right)$ do not change and are not reported.

	U(0,5)	U(−10,10)	$N\left(0,10^{2}\right)$	$N\left(0,10^{3}\right)$	$N\left(0,10^{4}\right)$
$\mathrm{KL}[\pi^{J}(.|y)||\pi_{1}]$	1.43	1.42	1.37	1.42	1.42
$\mathrm{KL}[\pi^{J}(.|y)||\pi_{2}]$	2.86	2.84	2.75	2.85	2.85
$\mathrm{KL}[\pi^{J}(.|y)||\pi_{3}]$	5.76	5.75	5.67	5.76	5.77
$\mathrm{KL}[\pi^{J}(.|y)||\pi_{4}]$	0.19	0.19	0.20	0.19	0.19
$\mathrm{KL}[\pi^{J}(.|y)||\pi^{J}]$	2.55	3.93	4.18	6.46	8.76
$m^{J}\left(y\right)$	1.16 × 10^−68^	2.91 × 10^−69^	5.65 × 10^−69^	2.26 × 10^−69^	7.33 × 10^−70^

**Table 4:** Comparison between experts based on KL divergences and marginal likelihoods. We report BF in favor of the row over the column and KL ratios for loss of information of the row over loss of information of the column.

	Expert 1		Expert 2		Expert 3		Expert 4	
	KL Ratio	BF	KL Ratio	BF	KL Ratio	BF	KL Ratio	BF
Expert 1	1	1	0.50	0.82	0.25	25.42	7.63	0.32
Expert 2	2.00	1.22	1	1	0.50	31.13	15.23	0.40
Expert 3	4.03	0.04	2.02	0.03	1	1	30.75	0.01
Expert 4	0.13	3.09	0.07	2.52	0.03	78.54	1	1

## References

[1] Gelman A., Carlin J.B., Stern H.S., Dunson D.B., Vehtari A., Rubin D.B. (2013). Bayesian Data Analysis.

[2] Lynch S.M. (2007). Introduction to Applied Bayesian Statistics and Estimation for Social Scientists.

[3] Zyphur M.J., Oswald F.L., Rupp D.E. (2013). Bayesian probability and statistics in management research. J. Manag..

[4] Bolsinova M., Hoijtink H., Vermeulen J.A., Béguin A. (2017). Using expert knowledge for test linking. Psychol. Methods.

[5] O’Hagan A., Buck C.E., Daneshkhah A., Eiser J.R., Garthwaite P.H., Jenkinson D.J., Oakley J.E., Rakow T. (2006). Uncertain Judgements: Eliciting Experts’ Probabilities.

[6] Zondervan-Zwijnenburg M., van de Schoot-Hubeek W., Lek K., Hoijtink H., van de Schoot R. (2017). Application and evaluation of an expert judgment elicitation procedure for correlations. Front. Psychol..

[7] Bousquet N. (2008). Diagnostics of prior-data agreement in applied Bayesian analysis. J. Appl. Stat..

[8] Kullback S., Leibler R.A. (1951). On information and sufficiency. Ann. Math. Stat..

[9] Cooke R. (1991). Experts in Uncertainty: Opinion and Subjective Probability in Science.

[10] Quigley J., Colson A., Aspinall W., Cooke R.M., Dias L.C., Morton A., Quigley J. (2018). Elicitation in the classical model. Elicitation.

[11] Walley R.J., Smith C.L., Gale J.D., Woodward P. (2015). Advantages of a wholly Bayesian approach to assessing efficacy in early drug development: A case study. Pharm. Stat..

[12] Fu S., Celeux G., Bousquet N., Couplet M. (2015). Bayesian inference for inverse problems occurring in uncertainty analysis. Int. J. Uncertain. Quantif..

[13] Fu S., Couplet M., Bousquet N. (2017). An adaptive kriging method for solving nonlinear inverse statistical problems. Environmetrics.

[14] Cohen J. (1960). A coefficient of agreement for nominal scales. Educ. Psychol. Meas..

[15] Koch G.G. (2004). Intraclass correlation coefficient. Encycl. Stat. Sci..

[16] Shrout P.E., Fleiss J.L. (1979). Intraclass correlations: Uses in assessing rater reliability. Psychol. Bull..

[17] Brier G.W. (1950). Verification of forecasts expressed in terms of probability. Mon. Weather Rev..

[18] Barons M.J., Wright S.K., Smith J.Q., Dias L.C., Morton A., Quigley J. (2018). Eliciting probabilistic judgements for integrating decision support systems. Elicitation.

[19] Kass R.E., Raftery A.E. (1995). Bayes factors. J. Am. Stat. Assoc..

[20] Liu C.C., Aitkin M. (2008). Bayes factors: Prior sensitivity and model generalizability. J. Math. Psychol..

[21] Wasserman L. (2000). Bayesian model selection and model averaging. J. Math. Psychol..

[22] Bernardo J.M. (1979). Reference posterior distributions for Bayesian inference. J. R. Stat. Soc. Ser. B Methodol..

[23] Irony T., Singpurwalla N. (1997). Noninformative priors do not exist: A discussion with jose m. bernardo. J. Stat. Inference Plan..

[24] Berger J.O., Bernardo J.M. (1989). Estimating a product of means: Bayesian analysis with reference priors. J. Am. Stat. Assoc..

[25] Bernardo J.M., Smith A.F. (1994). Bayesian Theory.

[26] Berger J.O., Bernardo J.M., Sun D. (2009). The formal definition of reference priors. Ann. Stat..

[27] Gelman A., Simpson D., Betancourt M. (2017). The prior can often only be understood in the context of the likelihood. Entropy.

[28] Kass R.E., Wasserman L. (1996). The selection of prior distributions by formal rules. J. Am. Stat. Assoc..

[29] Jeffreys H. (1946). An invariant form for the prior probability in estimation problems. Proc. R. Soc. Lond. Ser. Math. Phys. Sci..

[30] Jeffreys S.H. (1961). Theory of Probability.

[31] Jaynes E.T. (1982). On the rationale of maximum-entropy methods. Proc. IEEE.

[32] Yang R., Berger J.O. (1996). A Catalog of Noninformative Priors.

[33] Akaike H. (1973). Information theory as an extension of the maximum likelihood principle. Second International Symposium on Information Theory.

[34] Burnham K.P., Anderson D.R. (2002). Model Selection and Multimodel Inference: A Practical Information-Theoretic Approach.

[35] Morey R.D., Romeijn J.-W., Rouder J.N. (2016). The philosophy of Bayes factors and the quantification of statistical evidence. J. Math. Psychol..

[36] Raftery A.E. (1996). Approximate Bayes factors and accounting for model uncertainty in generalised linear models. Biometrika.

[37] Dirac P.A.M. (1947). The Principles of Quantum Mechanics.

[38] Barber D. (2012). Bayesian Reasoning and Machine Learning.

[39] Van de Schoot R., Griffioen E., Winter S., Bedford T., French S., Hanea A.M., Nane G.F. (2018). Dealing with imperfect elicitation results. Expert Judgement in Risk and Decision Analysis.

[40] Veen D., Stoel D., Zondervan-Zwijnenburg M., van de Schoot R. (2017). Proposal for a Five-Step Method to Elicit Expert Judgement. Front. Psychol..

[41] Fernández C., Steel M.F. (1998). On Bayesian modeling of fat tails and skewness. J. Am. Stat. Assoc..

[42] Plummer M. Rjags: Bayesian Graphical Models Using MCMC. Proceedings of the 3rd International Workshop on Distributed Statistical Computing (DSC 2003).

[43] Gelman A., Rubin D.B. (1992). Inference from iterative simulation using multiple sequences. Stat. Sci..

[44] Stan Development Team RStan: The R Interface to Stan. https://cran.r-project.org/web/packages/rstan/.

[45] Gronau Q.F., Singmann H. Bridgesampling: Bridge Sampling for Marginal Likelihoods and Bayes Factors. https://cran.r-project.org/web/packages/bridgesampling/.

[46] Deza M.M., Deza E. (2009). Encyclopedia of distances. Encyclopedia of Distances.

