# Correction: Veen, D.; Stoel, D.; Schalken, N.; Mulder, K.; Van de Schoot, R. Using the Data Agreement Criterion to Rank Experts’ Beliefs. *Entropy* 2018, *20*, 592

**DOI:** 10.3390/e21030307

**Published:** 2019-03-21

**Authors:** Duco Veen, Diederick Stoel, Naomi Schalken, Kees Mulder, Rens van de Schoot

**Affiliations:** 1 Department of Methods and Statistics, Utrecht University, 3584 CH 14 Utrecht, The Netherlands; 2 ProfitWise International, 1054 HV 237 Amsterdam, The Netherlands; 3 Optentia Research Focus Area, North-West University, Vanderbijlpark 1900, South Africa

## Abstract

Due to a coding error the marginal likelihoods have not been correctly calculated for the empirical example and thus the Bayes Factors following from these marginal likelihoods are incorrect. The corrections required occur in Section 3.2 and in two paragraphs of the discussion in which the results are referred to. The corrections have limited consequences for the paper and the main conclusions hold. Additionally typos in Equations, and, an error in the numbering of the Equations are remedied.

## 1. Corrections in Section 3.2

- Corrected Table 2

**Table:** 

	KL Divergence	DAC_d_	DAC_d_ Ranking	$m_{d}\left(y\right)\;\&\;m^{J}\left(y\right)$	$BF_{Jd}$	$BF_{Jd}$ Ranking
Expert 1	1.43	0.56	2	5.57 × 10^−68^	0.21	3
Expert 2	2.86	1.12	3	6.82 × 10^−68^	0.17	2
Expert 3	5.76	2.26	4	2.19 × 10^−69^	5.31	4
Expert 4	0.19	0.07	1	1.72 × 10^−67^	0.07	1
Benchmark	2.55	-	-	1.16 × 10^−68^	-	-
- Corrected Table 3

**Table:** 

	*U*(0,5)	*U*(−10,10)	*N*(0,10^2^)	*N*(0,10^3^)	*N*(0,10^4^)
$\mathrm{KL}[\pi^{J}(.|y)||\pi_{1}]$	1.43	1.42	1.37	1.42	1.42
$\mathrm{KL}[\pi^{J}(.|y)||\pi_{2}]$	2.86	2.84	2.75	2.85	2.85
$\mathrm{KL}[\pi^{J}(.|y)||\pi_{3}]$	5.76	5.75	5.67	5.76	5.77
$\mathrm{KL}[\pi^{J}(.|y)||\pi_{4}]$	0.19	0.19	0.20	0.19	0.19
$\mathrm{KL}[\pi^{J}(.|y)||\pi^{J}]$	2.55	3.93	4.18	6.46	8.76
$m^{J}\left(y\right)$	1.16 × 10^−68^	2.91 × 10^−69^	5.65 × 10^−69^	2.26 × 10^−69^	7.33 × 10^−70^
- Corrected Table 4

**Table:** 

	Expert 1		Expert 2		Expert 3		Expert 4	
	KL Ratio	BF	KL Ratio	BF	KL Ratio	BF	KL Ratio	BF
Expert 1	1	1	0.50	0.82	0.25	25.42	7.63	0.32
Expert 2	2.00	1.22	1	1	0.50	31.13	15.23	0.40
Expert 3	4.03	0.04	2.02	0.03	1	1	30.75	0.01
Expert 4	0.13	3.09	0.07	2.52	0.03	78.54	1	1

## 2. New Version of Section 3.2 Paragraphs 3–6

The results of Table 2 show that expert four provided the best prediction out of the experts, when using both the ${\mathrm{DAC}}_{d}{\;\mathrm{and}\;\mathrm{the}\;\mathrm{BF}}_{\mathrm{Jd}}.$ Experts one and two provided similar predictions concerning their tacit knowledge; they expected almost the same value for the location parameter; however, expert one was less certain about this prediction (see Table 1). As the prediction of the location was not entirely correct, the increased uncertainty of expert one means that this expert provided more plausibility to the regions of the parameter space that were also supported by the data. Here we see the difference between ${\mathrm{DAC}}_{d}{\;\mathrm{and}\;\mathrm{the}\;\mathrm{BF}}_{\mathrm{Jd}}$ arise as discussed in Section 2.3. Overconfidence ifs penalized more severely by the ${\mathrm{DAC}}_{d}$ and as such the conclusion on which expert would be preferred changes between experts one and two depending on which measure you use. When we look at the ${\mathrm{DAC}}_{d}$, in the case when $\pi^{J}\left(\theta \right)$ is the U(0,5) density, the additional penalization of the overconfidence even causes a different conclusion between experts one and two, namely, expert one is in prior-data agreement and expert two is in prior-data disagreement. For the ${\mathrm{BF}}_{\mathrm{Jd}}$ both are concluded to be in agreement with the data. Expert three provided a prediction that, to a large extent, did not support the same parameter space as the data. In fact, expert three provides a lot of support for regions of the parameter space that the data did not support. The discrepancy between expert three and the data was of such proportions that, besides expert two, we also concluded a prior-data disagreement to exist for expert three. If we had no information beforehand, except knowing the region within which the average turnover per professional could fall, we would have lost less information than by considering the predictions of experts two and three. The ${\mathrm{BF}}_{\mathrm{Jd}}$ differs from the ${\mathrm{DAC}}_{d}$ in the sense that when $\pi^{J}\left(\theta \right)$ is the U(0,5) density, the benchmark only outperforms expert 3.

From the sensitivity analyses of Table 3 we can find that the reference posterior remains quite stable and therefore the KL divergences for the experts do not change substantially; however, the changing KL divergence for the benchmark would shift the prior-data disagreement boundary. When $\pi^{J}\left(\theta \right)$ was the $N\left(0,10^{3}\right)$ or $N\left(0,10^{4}\right)$ density, expert three would no longer be in prior-data conflict, whilst prior-data disagreement for expert two was only concluded if $\pi^{J}\left(\theta \right)$ was the U(0,5) density. For the BF changing the benchmark also shifts the prior-data (dis)agreement boundary arbitrarily. In this case our decisions on prior-data (dis)agreement would only change for the $N\left(0,10^{4}\right)$ prior, where expert 4 would no longer be in prior-data disagreement. The sensitivity analysis showed that decisions on prior-data (dis)agreement might not be entirely reliable, whilst the ranking of experts remained stable.

Table 4 shows the results when we only compare experts on their KL divergences and their marginal likelihoods and we omit the benchmarks. We see the difference between the BF and the KL divergence ratios when we compare experts one and two. The differences arise from the more severe penalization of overconfidence by KL divergences compared to BF, as discussed in Section 2.3. Using KL divergence ratios we concluded that expert two had twice the amount of loss of information, whilst the BF even favors expert two over expert one with odds of 1.22. 

The results of the empirical study show a slight difference in the conclusions with regard to the ranking of the experts depending on which measure we used, ${\mathrm{DAC}}_{d}$ or ${\mathrm{BF}}_{\mathrm{Jd}}$. Both measures select the same expert as being the best. If decisions should be made concerning average turnover per professional, decision makers would be wise to consult expert four, as this expert seemed to have the best knowledge of the underlying factors driving these results.

## 3. New Version of Section 4 Paragraph 1

In this paper, we use both the BF and the DAC to rank experts’ beliefs when they are specified in the probabilistic form of prior distributions. When comparing the BF and the DAC, the limiting case example of Section 2.3 springs to mind. In the introduction, we stated that forecasting without specifying uncertainty would not make sense to us and, in that light, we would prefer to use a measure that would classify doing so as undesirable behavior and punish this extreme case. An example of this behavior can be seen in the empirical example where while using the BF we would favor expert two over expert one, however whilst using KL divergences, we would favor expert one over expert two. 

## 4. Nev Version of Section 4 Paragraph 3

One of the reasons for the sensitivity of the DAC to different choices for $\pi^{J}\left(\theta \right)$ can be seen by comparing the KL divergences of expert one and two of the empirical example. As a referee pointed out to us, KL divergences are tail sensitive and this can be seen in this comparison. Expert one is a little more uncertain and as such the tail of $\pi_{1}\left(\theta \right)$ overlaps somewhat more with $\pi^{J}\left(\theta |y\right)$ than the tails of $\pi_{2}\left(\theta \right)$. This leads to half the loss of information. One could deem this tail sensitivity to be undesirable and, with differently shaped prior distributions, this problem might become more pronounced. If it is deemed undesirable, one could favor using the BF, which actually favors expert two with odds of 1.22 over expert 1. Alternatively, an interesting area for future research could be to investigate the use of alternative divergence measures. A good starting point for finding alternative measures can be found in the Encyclopedia of Distances by Deza and Deza [46].

## 5. Corrections in Equations

In Section 2.3 [1] only there is a consistent mistake in the brackets involved in the KL functions. There is one “]” bracket too many. The new Equations read:

$${\mathrm{DAC}}_{2,\;d}^{J}=\frac{m^{J}\left(y\right)}{m_{d}\left(y\right)}\exp\left\{\mathrm{KL}[\pi^{J}\left(\cdot |y\right)||\pi_{d}\left(\cdot |y\right)]\right\}=\;{\mathrm{BF}}_{\mathrm{Jd}}\exp\left\{\mathrm{KL}[\pi^{J}\left(\cdot |y\right)||\pi_{d}\left(\cdot |y\right)]\right\},$$

$\exp\left\{\mathrm{KL}[\pi^{J}\left(\cdot |y\right)||\pi_{d}\left(\cdot |y\right)]\right\}$ and $\mathrm{KL}[\pi^{J}\left(\cdot |y\right)||\delta_{\theta_{0}}\left(\cdot \right)]$.

The second change in the Equations is in the numbering. The equations go from (3)–(5), (4) is skipped in the numbering. This made the numbering of the equations flawed after Section 2.2.1. This has been corrected.
